# Stromal cell derived factor-1alpha protects stem cell derived insulin-producing cells from glucotoxicity under high glucose conditions *in-vitro* and ameliorates drug induced diabetes in rats

**DOI:** 10.1186/1479-5876-11-115

**Published:** 2013-05-06

**Authors:** Muhammad Tariq, Muhammad Sharif Masoud, Azra Mehmood, Shaheen N Khan, Sheikh Riazuddin

**Affiliations:** 1 National Centre of Excellence in Molecular Biology, University of the Punjab, Lahore, Pakistan; 2 Current Affiliation: Department of Biotechnology, Mirpur University of Science and Technology, Mirpur, AK, Pakistan; 3 Current Affiliation: Department of Bioinformatics and Biotechnology, Government College University, Faisalabad 38000, Pakistan

**Keywords:** Diabetes mellitus, Mesenchymal stem cells, Differentiation, Preconditioning, SDF-1α

## Abstract

**Background:**

Diabetes mellitus is affecting more than 300 million people worldwide. Current treatment strategies cannot prevent secondary complications. Stem cells due to their regenerative power have long been the attractive target for the cell-based therapies. Mesenchymal stem cells (MSCs) possess the ability to differentiate into several cell types and to escape immune recognition *in vitro*. MSCs can be differentiated into insulin-producing cells (IPCs) and could be an exciting therapy for diabetes but problems like poor engraftment and survivability need to be confronted. It was hypothesized that stromal cell derived factor- 1alpha (SDF-1alpha) will enhance therapeutic potential of stem cell derived IPCs by increasing their survival and proliferation rate.

**Methods:**

Novel culture conditions were developed to differentiate bone marrow derived mesenchymal stem cells (BMSCs) into IPCs by using endocrine differentiation inducers and growth factors via a three stage protocol. In order to enhance their therapeutic potential, we preconditioned IPCs with SDF-1alpha.

**Results:**

Our results showed that SDF-1alpha increases survival and proliferation of IPCs and protects them from glucotoxicity under high glucose conditions *in vitro*. SDF-1alpha also enhances the glucose responsive insulin secretion in IPCs *in vitro.* SDF-1alpha preconditioning reverses hyperglycemia and increase serum insulin in drug induced diabetic rats.

**Conclusions:**

The differentiation of BMSCs into IPCs and enhancement of their therapeutic potential by SDF-1alpha preconditioning may contribute to cell based therapies for diabetes.

## Background

Diabetes mellitus (DM), a life threatening single-cell metabolic disorder, is defined by the presence of hyperglycemia due to damage or faulty pancreatic beta cells. Type 1 diabetes results from T-cell mediated autoimmune demolition of β-cells [1]. At least 285 million people were affected from diabetes in 2010 worldwide and this number is increasing day by day [2]. The treatment of the absolute insulin deficiency resulting from Type 1 diabetes is very challenging. Despite significant advances in the manufacture, modification and delivery of insulin, insulin therapy remains relatively risky and cannot prevent secondary complications. Islet-transplantation, the most successful treatment of DM, is hampered due to issues such as; severe shortage of pancreas donors, low islet isolation success rate, difficulty in maintaining an insulin-free status and the side effects of anti-rejection drugs [3,4]. Thus it is the need of hour to look for new strategies to generate pancreatic beta cells either *in vitro* or *in vivo*. An attractive approach for reversing diabetes is beta cell regeneration from adult stem cells. By using beta cells from a patient’s own adult stem cells, immunosuppression can be avoided.

Bone marrow derived mesenchymal stem cells (BMSCs) are able to differentiate into a number of different cell types of mesodermal lineages such as adipocytes, osteoblasts and other mesodermal cells [5,6]. MSCs can be differentiated into insulin producing cells (IPCs) and their possible therapeutic potential for diabetes depends upon this differential ability [7-9]. IPCs express the pancreatic β-cells developmental genes such as pancreatic and duodenal homeobox-1 (PDX-1) or functional genes such as insulin, and glucagon [8-11] and secrete insulin in response to glucose that reverses hyperglycemia in drug-induced diabetic mice [8,9,12]. The remedial use of MSCs is limited at present due to several problems such as poor engraftment, limited differentiation in host tissue [13] and differentiation of MSCs into unwanted lineages [14] by a variety of factors [15].

The enhancement of survival and proliferation of MSCs by growth factors has been extensively studied. The majority of growth factors is pleiotropic and changes motility, proliferation, morphogenesis and survival of cells. Glucose homeostasis is regulated by several polypeptides such as insulin, adiponectin, glucagon-like peptide–1 (GLP-1), and many others making them attractive candidates for the treatment of diabetes [16]. Many different strategies have been employed to induce the differentiation of IPCs from stem cells *in vitro.* These strategies have involved supplementation of differentiation medium with a variety of induction and growth factors, such as nourished ES cells with all-trans retinoic acid [17], betacellulin, activin A [18] and vitamin derivatives such as nicotinamide [19]. A number of studies have highlighted the role of GLP-1, a known anti-diabetic agent, in β cell development, function, proliferation and neogenesis [20,21]. Stromal cell-derived factor-1 (SDF-1), a chemokine known to express in stromal tissues in multiple organs, promotes β-cell survival in RIP-SDF-1 transgenic mice and MIN-6 and INS-1 clonal beta-cells by the activation of Akt signaling pathway and attenuates diabetes in streptozotocin (STZ) induced mice [22]. The activation of Akt signaling pathway have cell survival, cell proliferating and anti-apoptotic roles. SDF-1/CXCR4 axis modulates cell migration and cell survival during development and tissue remodeling [23]. Moreover, SDF-1α/CXCR4 is an obligatory component in the maintenance of pancreatic duct cell-survival, cell-proliferation and migration during pancreatic organogenesis and regeneration in pancreatic acinar cells [24]. The PI3K/Akt pathway is an important mediator of cell survival in many cell types such as beta cells [25-27] and PI3K/Akt signaling has been shown to play a central role in pancreatic regeneration [28]. To make use of the therapeutic potential of SDF-1α pre-conditioning in IPCs survival and proliferation, we hypothesized that SDF-1α pre-conditioning augments IPCs survival and may reverse hyperglycemia.

## Methods

### Animals care

The current study was carried according to the Guide for the Care and Use of Laboratory Animals published by the US National Institutes of Health (NIH Publication No. 85–23, revised 1996). All animals were treated according to the procedures approved by the Institutional Review Board (IRB) at the National Center of Excellence in Molecular Biology, Lahore, Pakistan.

### Animals

Inbred Sprague–Dawley (SD) rats, aged 8–16 weeks, were used in this study as streptozotocin can easily cause diabetes in these animals [29]. Rats were placed in a special room where the temperature was kept at 22°C and the light was on a 12 hr dark / light cycle throughout the year.

### Isolation and culture of rat BM-derived MSCs

Rat BMSCs were isolated and cultured as previously described [30]. In brief, bone marrow was isolated from the femur and tibia of 3–4 months old SD rats. After removing epiphyses and gaining access to the marrow cavities, whole BM was flushed out from bones. The resulting cell suspension was centrifuged and the cell pellet was re-suspended in DMEM-LG supplemented with 10% fetal bovine serum (FBS) and 1% antibiotic (100 μg/ml streptomycin and 100 U/ml penicillin). The cells were plated in tissue culture flask and placed in incubator having 5% CO_2_ and 95% air at 37°C. The medium was then changed after every three days. When cells were 80% confluent, they were sub-cultured in a ratio 1:3.

### Differentiation of BMSCs into IPCs

When BMSCs at 3rd passage were 70%-80% confluent, they were differentiated into insulin producing cells (IPCs) by a three stage protocol as follows. Stage 1: The cells were induced with DMEM-LG containing 0.5 mM/L 2-mercaptoethanol, 10 mM/L nicotinamide and 5% FBS for 2 days. Stage 2: The pre-induced cells were further treated with serum free DMEM high glucose (DMEM-HG) medium containing 0.5 mM/L 2-mercaptoethanol, 10 mM/L nicotinamide and 10 ng/ml activin A for 10 hours. Stage 3: The cells were cultured for an additional 8 days in new DMEM-HG medium containing 20 ng/ml β-fibroblast growth factor (bFGF, Sigma), 20 ng/ml epidermal growth factor (EGF, Sigma), 2% B27 (Gibco), 2 mM/L L-glutamine (Hyclone Laboratories), 10 mM/L nicotinamide and 50 ng/ml GLP-1.

### Identification of IPCs by dithizone (DTZ) staining

Presence of IPCs in culture can be identified with Zn-chelating agent dithizone which binds with free Zn^2+^ present in these cells. The stock solution was prepared by dissolving 50 mg of DTZ (Merck) in 5 ml DMSO and stored at −20°C until used. The working solution of DTZ was prepared by bringing stock solution to room temperature. 10 μl working DTZ solution was added to culture medium and incubated for 30 min at 37°C. The crimson-red-stained clusters were observed under a microscope.

### Immunocytochemical analysis for pancreatic developmental markers in IPCs

To confirm the differentiation of BMSCs into IPCs, cultured IPCs were incubated with primary antibodies specific to insulin, Ngn3 and Pdx-1 and then treated to respective secondary antibodies. Nuclei were counter-stained with DAPI. At least six randomly high power fields were selected from each slide. Fluorescent images were obtained with an Olympus IX 51 and BX 61 microscopes equipped with digital cameras (Olympus).

### RT-PCR analysis for expression of genes of pancreatic development in IPCs

Total cellular RNA was extracted from BMSCs, IPCs and pancreatic tissue by using TRIZOL reagent (Invitrogen). cDNA was synthesized using total RNA by Revert Aid H Minus first strand cDNA synthesis kit (Fermentas; Cat# K1632). Following PCR conditions were used: 1 cycle at 94°C for 4 min: 35 cycles at (94°C for 45 s, annealing temp for 45 s and 72°C for 60 s) and 1 extended cycle at 72°C for 10 min. The PCR products were size-fractionated by 2.0% agarose gel electrophoresis. The oligonucleotide sequences specific for the selected genes are presented in Table 1.

**Table 1:** Primer sequences of various genes

** Genes**	**Primer sequences**	
Insulin 1	Forward	5’-CAGTTGGTAGAGGGAGCAG-3’
	Reverse	5’-CAGTTGGTAGAGGGAGCAG-3’
Insulin 2	Forward	5’-TCATCCTCTGGGAGCCCCGC-3’
	Reverse	5’-AGTTGCAGTAGTTCTCCAGT-3’
Ngn3	Forward	5’-CTTCACAAGAAGTCTGAGAACACCAG-3’
	Reverse	5’-CTGCGCATAGCGGACCACAGCTTC-3’
Pdx-1	Forward	5’-GGTGCCAGAGTTCAGTGCTAA-3’
	Reverse	5’-CCAGTCTCGGTTCCATTCG-3’
Nkx 6.1	Forward	5’-ATGGGAAGAGAAAACACACCAGAC-3’
	Reverse	5’-TAATCGTCGTCGTCCTCCTCGTTC-3’
Nkx 6.2	Forward	5’-AGAAAGGTATGGAGGTGACG-3’
	Reverse	5’-CTGTACTGGGCGTTGTATTG-3’
β-Actin	Forward	5’-GCTGTGTTGTCCCTGTATGC-3’
	Reverse	5’-GAGCGCGTAACCCTCATAGA-3’

### Preconditioning of IPCs

At day 11, BMSCs derived IPCs were preconditioned with SDF-1α. IPCs were incubated in normal DMEM-HG containing 50 ng/ml GLP-1, 10% FBS and 50 ng/ml SDF-1α for 48 hours in CO_2_ incubator and rinsed for 10 min with normal medium for further *in vitro* and *in vivo* studies.

### *In vitro* experiments

Three experimental groups were assigned as follows: (I) Normal BMSCs at 3rd passage; (II) Non-preconditioned IPCs incubated in normal DMEM-HG containing 50 ng/ml GLP-1 and 10% FBS; (III) SDF-1α preconditioned IPCs incubated in normal DMEM-HG containing 50 ng/ml GLP-1, 10% FBS and 50 ng/ml SDF-1α. All groups were incubated for 48 hours in CO_2_ incubator and rinsed for 10 min with normal medium. To compare the effect of different glucose concentrations on above mentioned groups, a total of 2 × 10^4^ cells/well were cultured in 24-well plates in the DMEM containing 5.5 mM/L, 17 mM/L and 33 mM/L glucose for 2 days.

### Measurement of lactate dehydrogenase (LDH) and cell viability

The cell viability was evaluated after treating with different concentrations of glucose by the trypan blue dye-exclusion method. Cell supernatant was analyzed for LDH activity using LDH based *in vitro* toxicological kit TOX7 (Sigma) and taking absorbance at 490 nm and a reference wavelength of 690 nm to obtain sample signal (OD_490_-OD_690_).

### Cell proliferation assay

The cells treated with different glucose concentrations were incubated for 2–3 hrs with 40 μl/well MTT (3-(4, 5-Dimethylthiazol-2-yl)-2, 5-diphenyltetrazolium bromide) reagent (Sigma-Aldrich) having concentration of 5 mg/ml. The medium was aspirated and the cells were suspended in dimethyl sulfoxide. Absorbance of the formazan product which is directly proportional to the number of living cells was measured by an ELISA plate reader at 570 nm and a reference wavelength of 630 nm to obtain sample signal (OD_570_-OD_630_).

### ELISA for insulin

To determine whether SDF-1α preconditioning causes an increase in insulin release from IPCs, an enzyme-linked immunoassay (ELISA) was used to quantify insulin levels in various groups after incubating with different glucose concentrations for 2 days. Cells were incubated with freshly prepared Krebs Ringer bicarbonate HEPES (KRBH) buffer supplemented with 3.8 mM/L glucose for 2 hrs at 37°C. Then KRBH buffer supplemented with 5.5 mM/L, 17 mM/L and 33 mM/L glucose was added to tissue culture plates and incubated for 3 hrs at 37°C. Supernatant was collected and insulin released was determined by ELISA. The absorbance was measured at 490nm and evaluated. The results were compared with a standard curve constructed with murine insulin (each assay carried out in triplicate for each group).

### *In vivo* experiments

#### Induction of diabetes

Diabetes was induced in 20 female SD rats at 8–16 weeks of age by intraperitoneal injection of 40 mg/kg of streptozotocin (STZ) in cold sodium citrate buffer (pH 4.5). Control rats were treated with citrate buffer only. Blood glucose levels were measured by using Roche ACCU-CHEK glucometer. Stable blood glucose levels ranging between 300–520 mg/dl developed in rats one week later while blood glucose levels of normal rats were in range of 75–125 mg/dl.

#### PKH26-labelling of cells

Prior to transplantation in the kidney capsule, IPCs were labeled with PKH26 (Sigma, Product no.: PKH26-GL) according to manufacturer’s protocol. This lipophilic dye PKH26 binds irreversibly to the cell membranes and serves an important marker for tracking stem cells in the transplanted tissue.

#### Transplantation

Diabetic rats were divided into three groups (n = 12) after showing consistent hyperglycemia (blood glucose > 300 mg/dl) for at least three measurements. The diabetic rats of 1st group received IPCs pre-conditioned with SDF-1α, 2nd group received IPCs only and 3rd group was injected with serum free medium. Rats were anesthetized, shaved from left dorsal side and cleaned. A small incision was made on the left flank of the rat and the kidney was exposed. A total of 3×10^6^ labeled cells were transplanted into the left kidney capsule at three different sites.

#### Measurement of blood glucose and serum insulin

The blood glucose levels were monitored at every third day by using AccuChek glucometer for 30 days after transplantation. The blood samples from transplanted rats were collected at day 7, 14, 21 and 28. Serum was isolated from these samples and insulin was measured by ELISA.

#### Organ procurement and processing

Rats were euthanized at 28 days after cell transplantation. For cryo sectioning, the excised kidney and pancreatic tissues were embedded in Tissue-Tek OCT (Sakura Torrance, CA, USA). 5 um thick tissue sections were cut with the help of cryostat (Microm) at -20C°.

#### Homing of transplanted cells

To assess the homing of transplanted IPCs, the sections were prepared from frozen tissues. Sections were examined under fluorescent microscope Olympus BX61 using standard filter setup for TRITC (PKH26). The stained sections were digitally imaged using a computerized image-analysis system. The number of PKH 26 positive cells was counted in six randomly selected areas.

#### Immunohistochemical analysis for Ki-67 for determination of proliferating cells

The proliferating cells in pancreata of diabetic rats after transplantation were seen by staining with immPRESS Reagent (Vector Labs) using manufacturer’s protocol. Briefly, air dried, acetone fixed frozen sections were washed with PBS and incubated for 20 min with 2.5% normal horse blocking serum. Sections were incubated with primary antibodies specific to Ki-67 and treated with ImmPRESS reagent. Sections were incubated in peroxidase substrate solution until sufficient stain developed. Nuclei were counter-stained with eosin. At least six randomly high power fields were selected from each slide. Fluorescent images were obtained with an Olympus BX61 microscope equipped with digital cameras (Olympus).

#### Statistical analysis

Experiments were performed in quadruplicate and repeated at least three times. All values are expressed as mean ± standard error of the mean (SEM). Statistical significance was analyzed using one way ANOVA followed by Bonferroni testing through Graphpad Prism 5 software. A *p*-value ≤ 0.05 was considered statistically significant.

## Results and discussion

### *In vitro* study

### Differentiation of BMSCs into beta like IPCs

**Figure 1:**
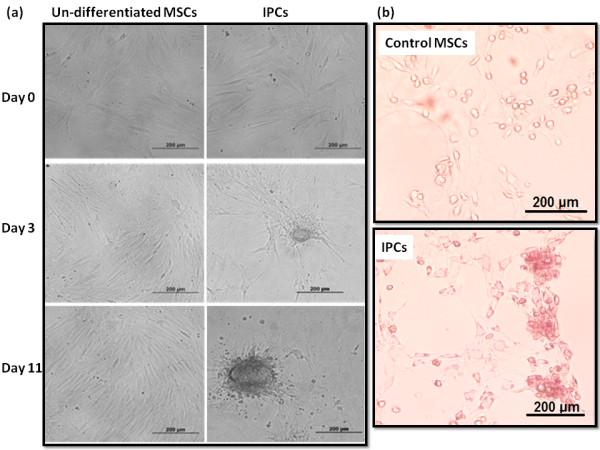
**Morphology and dithizone staining of differentiation of MSCs into IPCs. a)** Morphological changes during differentiation of MSCs into IPCs days 0, 3 and 11. Undifferentiated (left) and differentiated (right). Formation of islet-like clusters in differentiated group started at day 3 and matured towards day 11. **b)** Detection of Insulin producing cells after differentiation. At day 11, cells were stained with zinc-chelating agent, dithizone. Differentiated MSCs-derived IPCs at day 11 were distinctly stained crimson-red by DTZ.

**Figure 2:**
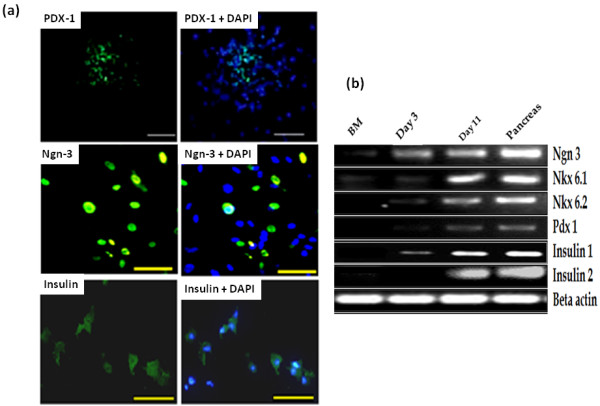
**Expression of pancreas specific hormones and transcription factors in differentiated IPCs at day11. a)** Immunofluorescence analysis in differentiated IPCs at day11 of Pdx-1 (10X), Ngn3 (20X) and Insulin (20X). **b)** Reverse transcriptase analysis. **Lane 1)** BM-derived MSCs at passage-3 before the start of treatment at day 0 **Lane 2)** BM-derived MSCs after pre-induction after stage-2 at day 3 **Lane 3)** Differentiated BM-derived MSCs after stage-3 at day 11 **Lane 4)** Pancreas as positive control.

At day 5 of induction, BMSCs started to form sphere-shaped clusters which attained their maximum size and maximal number at day 11 while control BMSCs which were not induced did not show any cluster formation (Figure 1a). Many cells in the culture induced with different reagents and growth factors stained crimson red with DTZ (Figure 1b) indicating the differentiation of BMSCs into IPCs. Control BMSCs were not stained. The culture induced with different reagents and growth factors expressed transcripts of insulin-I and insulin-II along with transcription factors Pdx-1, Ngn3, Nkx 6.1 and Nkx 6.2 (Figure 2b) at the completion of treatment. Immunostaining of induced cultured cells showed many insulin positive cells (Figure 2a) while the nuclei of differentiated IPCs expressed transcription factors Ngn3 and PDX-1 (Figure 2a). Treatment of BMSCs with insulin-promoting factors such as nicotinamide, high glucose induction and growth factors such as activin A and GLP-1 differentiate them into IPCs. Nicotinamide, a poly(ADP-ribose) synthetase inhibitor, is a well-known inducer to differentiate stem cells into IPCs and protect cells from glucotoxicity induced by exposure to high glucose [9,12,19,31]. Tang *et al*., (2004) considered high glucose concentration as a potent inducer for pancreatic islet differentiation [32]. But Sun *et al*., (2007) reported that high glucose alone could not induce bone marrow-derived MSCs to IPCs [9]. Activin A, a member of the transforming growth factor-beta (TGF-β) super-family, regulated neogenesis of β-cells *in vivo*[33] and induced ESCs into pancreatic β-cells [18]. GLP-1, an incretin hormone, used to convert intestinal epithelial cells into IPCs [34] and also differentiated BMSCs into IPCs [35]. Neshati *et al*., (2010) indicated that treatment of MSCs with high glucose, nicotinamide and 2-mercaptoethanol differentiated them into IPCs [36]. In the present study, we combined the previously used endocrine differentiation inducers in a novel way to differentiate BMSCs into IPCs.

The pancreas develops under a cascade of gene activation events controlled by transcription factors including PDX-1, Ngn3, NeuroD1, PAX-6, PAX-4, Nkx2.2, Nkx6.1 and so on [37,38]. Nkx-6.1 is exclusively expressed in the islets of Langerhans in differentiating and mature beta-cells. The presence of PDX-1 is required for the expression of Nkx-6.1 as well as other pancreatic beta cell specific genes, including insulin and both Nkx 6.1 and Nkx 6.2 activity is required for α- and beta-cell development in the pancreas [39]. Nkx 6.1 regulates the expression of Nkx 6.2 [39]. Therefore, *in vitro* differentiated IPCs should express genes of the pancreatic developmental transcription factors mentioned above along with the genes for insulin I and insulin II.

In this study, the expression of PDX1 observed at day 3 (Figure 2b) may have initiated a cascade of events that leads towards insulin transcription as PDX-1 controls the transcription of endocrine progenitor cells. PDX-1 contributes to specification of endocrine progenitors both by regulating expression of Ngn3 directly and by participating in a cross-regulatory transcription factor network during early pancreas development [40]. Following PDX-1 gene activation, the pancreatic transcriptional activation of genes such as Ngn3 occurs [41,42]. In our study, Ngn3 stably expressed at day 11 (Figure 2b). Ngn3 is required for the development of the four endocrine cell lineages (alpha, beta, delta and PP) of the pancreas [43]. In our study both Nkx 6.1 and Nkx 6.2 were expressed during the differentiation of BMSCs into IPCs. The expression of the transcripts of the major pancreatic hormones insulin-1 and insulin-2 further confirmed the differentiation of IPCs. The gene expression pattern observed during differentiation (Figure 2b) was similar to that of pancreatic tissue.

### SDF-1α preconditioning enhances cell viability and proliferation of IPCs under high glucose conditions

**Figure 3:**
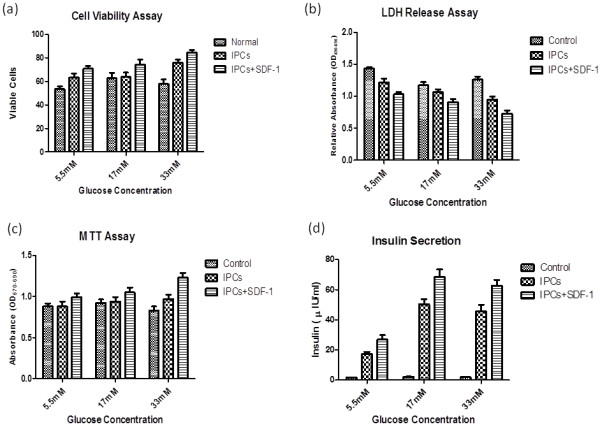
**Effect of SDF-1α preconditioning on IPCs under different glucose concentrations *in vitro*. a)** The number of viable cells is plotted in relevance with normal BM-derived MSCs. There is a marked increase in cell survivability in pre-conditioned group than control and non pre-conditioned groups (n = 6)**. b)** Cyto-protective effect of SDF-1α pre-conditioning on IPCs. Pre-conditioned IPCs showed low level of LDH compared to the control and non pre-conditioned groups indicating less cellular injury. **c)** Effect of SDF-1α pre-conditioning on cell proliferation under different glucose concentrations. Pre-conditioned IPCs showed higher rate of proliferation as compared to the control and non pre-conditioned groups. **d)** Glucose responsive insulin release by SDF-1α pre-conditioned IPCs. The IPCs were exposed to either 5.5 mM/L, 17 mM/L or 33 mM/L glucose. Insulin released from the IPCs upon glucose induction was determined by ELISA. The experiments were performed in triplicates and repeated three times. All values were expressed as mean ± SEM.

This study demonstrated for the first time the effect of SDF-1α pre-conditioning on IPCs *in vitro* as well as *in vivo*. Differentiated IPCs were pre-conditioned with SDF-1α. Cell viability evaluated by trypan blue exclusion assay showed marked increase in viable cells in SDF-1α preconditioned group compared with the control and non-preconditioned group (Figure 3a). To assess cell proliferation in the differentiated IPCs after pre-conditioning with SDF-1α, MTT assay was performed. IPCs pre-conditioned with SDF-1α showed better proliferation (1.21 ± 0.041) than non-preconditioned IPCs (1.05 ± 0.027) under high (33 mM/L) glucose concentration (Figure 3c). Our results demonstrated that SDF-1α pre-conditioning of IPCs significantly enhanced cell viability as compared to control BMSCs and non-preconditioned IPCs under low as well as high glucose concentrations (Figure 3a) and showed better proliferation under different glucose concentrations (Figure 3c). Previous studies showed that SDF-1α promotes pancreatic beta-cell survival by the activation of Akt signaling pathway and has cell proliferating, cell survival, anti-apoptotic roles [22], chemotactic properties and facilitates the homing of MSCs in various tissues [44,45].

### SDF-1α preconditioning protects IPCs from glucotoxicity

These findings were further confirmed by LDH release assay which revealed that preconditioning of IPCs with SDF-1α extensively lessened the cyto-pathic effects induced by high glucose *in vitro* (Figure 3b). This indicated that pre-conditioning reduces glucotoxicity caused by high glucose *in vitro.* Therefore, these results are in accordance with other studies conducted to explore biological effects of SDF-1α in development of pancreas and in cell based therapies for other organs [41,44,45]. Futhur more, pre-conditioning of MSCs with growth factors etc. plays a major role in augmenting resistance of MSCs to stress due to injury or disease *in vitro* or *in vivo*[46].

### SDF-1α preconditioned IPCs are glucose responsive

The pancreatic β-cells possess the ability to regulate the secretion of insulin in a glucose dependent manner. The glucose-responsive insulin release by SDF-1α pre-conditioned IPCs and non-preconditioned IPCs was analyzed *in vitro* by ELISA after exposing to high glucose. Both BM-derived non-preconditioned IPCs and SDF-1α pre-conditioned IPCs secreted insulin in response to high glucose challenge in a glucose-regulated manner but SDF-1α pre-conditioned IPCs distinctly showed higher rate of insulin release (67.3 ± 4.9 Vs 60 ± 5.4) and (116.6 ± 5.9 Vs 85.7 ± 6.5) at 17 and 33 mM/L glucose concentrations respectively (Figure 3d) indicating that SDF-1α pre-conditioning had positive impact on the glucose dependent insulin release. Control BMSCs cultured in normal medium showed no significant release of insulin in different glucose concentrations.

### *In vivo* studies

#### Reversal of hyperglycemia in STZ-induced diabetic rats

**Figure 4:**
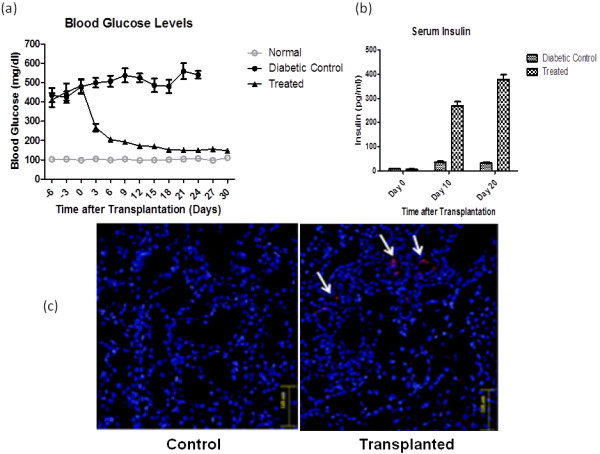
**Functional analysis after SDF-1α preconditioned IPCs transplantation in vivo. a)** Blood glucose levels after SDF-1α Pre-conditioned IPCs transplantation. Glucose levels were monitored by tail-vein blood at under random conditions. **b)** Serum Insulin Levels after Transplantation of SDF-1α Pre-conditioned IPCs. The serum insulin measured by ELISA of serum samples at days mentioned. The day 0 indicates the day of cell transplantation. All values are expressed as mean ± SEM. **c)** Homing of transplanted SDF-1α Pre-conditioned IPCs in left Kidney at d 10 in control diabetic rats and SDF-1α preconditioned IPCs transplanted rats.

To observe the role of SDF-1α pre-conditioning on IPCs *in vivo*, 3 × 10^6^ SDF-1α pre-conditioned IPCs were transplanted in left kidney capsule of the STZ-induced diabetic rats (n = 8). The control group (n = 8) was injected with serum free medium only. The blood glucose level of transplanted rats was monitored for 28 days after transplantation. The results showed that the glucose levels in the rats transplanted with SDF-1α pre-conditioned IPCs (173 ± 10.4 mg/dl) decreased significantly (Figure 4a) as compared to diabetic control group (482 ± 26.8 mg/dl). The decrease in glucose levels in transplanted group was significant. The control diabetic rats remained hyperglycemic throughout the study period.

#### Increase in serum insulin levels of transplanted diabetic rats

The functionality of SDF-1α pre-conditioned IPCs *in vivo* was assessed by serum insulin level measurement by ELISA. The results demonstrated that the insulin levels in the SDF-1α pre-conditioned IPCs group was increased from (0.317 ± 0.01) at day 10 to (0.47 ± 0.024) at day 20. This increase was significant as compared to diabetic control group (0.032 ± 0.009) at day 10 and (0.027 ± 0.024) at day 20 (Figure 4b).

#### SDF-1α preconditioning enhances homing of IPCs in diabetic kidney

The homing of transplanted IPCs was assessed by tracking PKH-26 labeled IPCs in left kidney, the site of transplantation. The results showed several PKH-26 labeled IPCs in the sections of left kidneys of recipient rats (Figure 4c).

#### Ki-67 staining for the determination of proliferating cells

**Figure 5:**
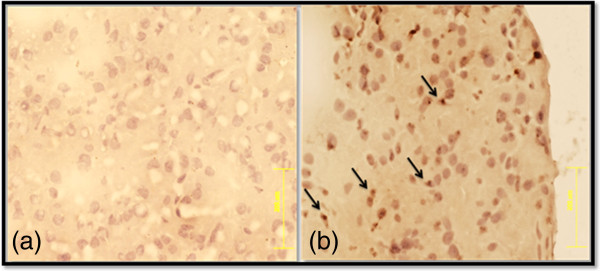
**The Ki-67 staining of pancreatic sections of rats transplanted with SDF-1α preconditioned IPCs.** Proliferating cells stained with Ki-67 in pancreas after transplantation of SDF-1α preconditioned IPCs in control diabetic rats **(a)** and SDF-1α preconditioned IPCs transplanted rats **(b)**.

The Ki-67 staining of pancreatic sections of rats transplanted with SDF-1α pre-conditioned IPCs revealed many Ki-67 positive cells in pancreas. There were no Ki-67 positive cells in the pancreatic sections of control diabetic rats which received only serum free medium (Figure 5). This confirms the presence of proliferating cells in rats transplanted with SDF-1α preconditioned IPCs.

## Conclusions

Therapeutic potential of stem cells has made stem cell research one of the most promising fields in biomedical research. There is a growing concern to find alternative sources of β-cells to cure diabetes. MSCs have emerged as a new cell therapeutic tool in regenerative medicine. We in this study with slight difference from many others [9,31-33] reported that BMSCs can be differentiated into IPCs but this study demonstrated for the first time the effect of SDF-1α pre-conditioning on IPCs *in vitro* as well as *in vivo*. SDF-1α enhances their survival and proliferation, lowers apoptosis in vitro and reverses STZ-induced diabetes in rats. The results demonstrated that the insulin secreted by SDF-1α pre-conditioned IPCs after induction with high glucose was glucose responsive. *In vivo* study demonstrated reversal of hyperglycemia and an increase in serum insulin levels in the rats transplanted with SDF-1α pre-conditioned IPCs.

## Abbreviations

MSCs: Mesenchymal stem cells; IPCs: Insulin producing cells; SDF-1α: Stromal cell derived factor-1alpha; DM: Diabetes mellitus; BMSCs: Bone marrow derived mesenchymal stem cells; PDX-1: Pancreatic and duodenal homeobox-1; GLP-1: Glucagon-like peptide–1; SD: Sprague–Dawley; EGF: Epidermal growth factor; DTZ: Dithizone; STZ: Streptozotocin; LDH: Lactate dehydrogenase; KRBH: Krebs Ringer bicarbonate HEPES.

## Competing interests

All authors confirmed that, “no competing interests exist”.

## Authors’ contributions

MT conceived the idea, participated in the design of study, performed culturing and *in vitro* treatments, participated in *in vivo* experiments, executed statistical analysis and drafted the paper. MSM participated in transplantation and surgical procedures. AM analyzed the *in vitro* data and helped to draft the manuscript. SNK participated in study design and proofreading the manuscript and SR contributed in study design, funding and final approval of the manuscript. All authors read and approved the final manuscript.
